# A Low-Cost Multi-Parameter Water Quality Monitoring System

**DOI:** 10.3390/s21113775

**Published:** 2021-05-29

**Authors:** Arif Ul Alam, Dennis Clyne, M. Jamal Deen

**Affiliations:** Department of Electrical and Computer Engineering, McMaster University, Hamilton, ON L8S 4K1, Canada; alamau@mcmaster.ca (A.U.A.); clyned@mcmaster.ca (D.C.)

**Keywords:** electrochemical sensors, free chlorine, heavy metal, pH, pharmaceutical contaminants, potentiostat, temperature sensor, water quality monitoring, water sensors

## Abstract

Multi-parameter water quality monitoring is crucial in resource-limited areas to provide persistent water safety. Conventional water monitoring techniques are time-consuming, require skilled personnel, are not user-friendly and are incompatible with operating on-site. Here, we develop a multi-parameter water quality monitoring system (MWQMS) that includes an array of low-cost, easy-to-use, high-sensitivity electrochemical sensors, as well as custom-designed sensor readout circuitry and smartphone application with wireless connectivity. The system overcomes the need of costly laboratory-based testing methods and the requirement of skilled workers. The proposed MWQMS system can simultaneously monitor pH, free chlorine, and temperature with sensitivities of 57.5 mV/pH, 186 nA/ppm and 16.9 mV/°C, respectively, as well as sensing of BPA with <10 nM limit of detection. The system also provides seamless interconnection between transduction of the sensors’ signal, signal processing, wireless data transfer and smartphone app-based operation. This interconnection was accomplished by fabricating nanomaterial and carbon nanotube-based sensors on a common substrate, integrating these sensors to a readout circuit and transmitting the sensor data to an Android application. The MWQMS system provides a general platform technology where an array of other water monitoring sensors can also be easily integrated and programmed. Such a system can offer tremendous opportunity for a broad range of environmental monitoring applications.

## 1. Introduction

Water quality monitoring is vital for water safety determination and associated public health [1,2,3,4,5]. Water quality parameters are decided by mutually dependent chemical, physical, and microbial features. Typical water quality parameters include pH, free chlorine, conductivity, dissolved oxygen, turbidity, and bacterial contamination [6,7]. Some of the most important but simple water quality parameters are pH, free chlorine and temperature due to their direct relationship with their water disinfection efficiency, as well as their influence to other parameters [8]. Although many water treatment plants use chloramines as disinfectants, the use of free chlorine is still the most common method for disinfection in isolated and resource-limited areas [9]. The presence of organic micropollutants such as bisphenol A (BPA) in water is another emerging water problem due to industrial effluents, and widespread use and disposal of plastics in the environment [10]. Standard water quality monitoring systems are complex, time-consuming and expensive due to transport of samples, use of sophisticated equipment and trained personnel [8]. Many conventional systems require independent collection of samples and assessment, and fail to deliver water quality parameters in real-time [11]. Real-time water quality monitoring technologies have made considerable advancement in recent years [11]. However, these technologies have limited applications monitoring source water or the operation of water treatment plants. Also, the high expenses related with the equipment, maintenance and calibration of water sensors have prevented them from being used in large distribution systems and for end-users. Furthermore, most of these sensor systems measure only one parameter at a time [12,13,14]. Therefore, it is critical to monitor key water quality parameters with high sensitivity and accuracy in a reliable and easy-to-use manner, which can be achieved with a multi-parameter sensing platform.

Recently, integrated wearable sensing systems were developed to monitor sweat biomarkers and the treatment of diabetes [15,16,17]. These systems provide accurate sensing and measurement data with high spatial and temporal resolution. They can also perform simultaneous and on-site signal processing with their integrated electronics. However, most of these systems are complex and expensive. Thus far, many integrated water quality sensor systems have been reported for pH, dissolved oxygen, conductivity, temperature, and microorganism including bacterial and organic nutrients/metabolites monitoring [18,19,20,21,22]. Some of these parameters, including pH and temperature, were identified as Key Performance Indicators (KPI) towards developing a Smart Water Quality Monitoring System (SWQMS) [23]. Smart and cost-effective water quality monitoring systems have been demonstrated, that utilize Internet of Things (IoT) [24] and Information and Communications Technology (ICT) [25] to enable connections between different sensors and devices. Currently available integrated sensing systems typically include free chlorine. Standard free chlorine sensing system involve the use of *N*,*N*′-diethyl-p-phenylenediamine (DPD), which is an optical absorption-based method that is not very user-friendly, susceptible to human error and costly. On the other hand, amperometric free chlorine sensors require frequent calibration as the stability of the sensing electrodes degrades over time. Moreover, BPA is commonly measured by conventional analytical techniques based on chromatography methods such as liquid chromatography, liquid chromatography–mass spectrometry, and immunoassays, which require skilled personnel and expensive equipment. Therefore, a new design approach is required to develop an integrated, low-cost and accurate sensing system where many of the water quality parameters can complement one another to evaluate the overall quality of the water. Such an integrated sensing system, that can monitor pH, free chlorine (Cl), temperature and organic micropollutants for hand-held applications such as drinking water and swimming pool water monitoring, needs to be developed.

In this research, we develop a Multi-Parameter Water Quality Monitoring System (MWQMS) that can simultaneously measure pH, free Cl concentration, and temperature, and electrochemically measure (i.e., linear sweeping voltammetry) BPA in drinking water. The proposed MWQMS system is designed to accomplish a seamless interconnection between sensing electrodes, sensor signal transduction, signal conditioning, data processing and low-power wireless transmission of the sensors signals to a smartphone interface. The MWQMS system also integrates the potentiometric, amperometric and voltametric sensors, providing a more ubiquitous platform for different electrochemical sensing approaches. A custom-designed, small-form-factor and multi-layered printed circuit board (PCB) was designed with off-the-shelf integrated-circuit components, microcontrollers and open-source software programming. Also, low-cost electrochemical sensors were fabricated on glass substrates that were connected to the PCB readout circuit board. The MWQMS system has significantly reduced the complexity and associated high costs of the sensor as well as the design of the electronic data acquisition system.

## 2. Materials and Methods

### 2.1. Fabrication of the Sensors

**Figure 1:**
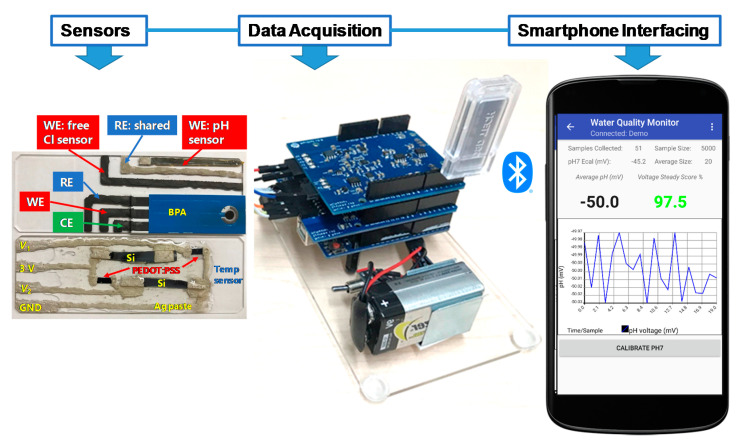
Photograph of MWQMS sensors on two glass slides. The upper one contains the pH, free Cl and BPA sensors, and the lower one contains the temperature sensor. The photograph also shows Arduino Uno microcontroller-based printed circuit boards together with a Bluetooth transceiver for wireless connection and a custom-developed Android app.

The fabrication processes of the array of electrochemical sensors for the MWQMS were based on our recent study [26]. In ref. [26], we developed materials synthesis, sensor fabrication and their performance characterizations. In contrast, in this study, we developed a complete sensing system through the integration of the sensors with our custom-designed readout system, smartphone-controlled operation and real samples analysis. Although the sensors were developed and calibrated in our previous study [26], the sensors were calibrated again in this study with our custom-designed readout circuit and smartphone application in order to validate the sensors performance with the integrated MWQMS system. In brief, the pH sensor was a potentiometric sensor that was fabricated with Palladium (Pd) ink on polyimide film substrate. The free Cl sensor was an amperometric sensor that was fabricated with a carbon-based electrode with subsequent electrochemical modification by ammonium carbamate. The reference electrode was made of Ag/AgCl with commercial Ag/AgCl paste. The temperature sensor was based on resistance-change that was fabricated with *p*-type Si and poly(3,4-ethylenedioxythiophene) polystyrene sulfonate (PEDOT:PSS) film in a Wheatstone bridge configuration. The sensors for the organic micropollutants (i.e., BPA) was fabricated by attaching a commercial paper-based screen-printed carbon electrode (SPE) onto the substrate containing the pH and free Cl sensors, followed by modification with graphene oxide (GO) and β-cyclodextrin functionalized multi-walled carbon nanotubes (GO-MWCNT-βCD). The synthesis of the chemically functionalized MWCNT-βCD is based on our previous study [27]. The MWCNT-βCD is further mixed with GO with a 1:1 ratio, to enhance the electrochemical sensing performance towards detecting BPA. The SPE consists of a carbon-based counter electrode and an Ag/AgCl based printed reference electrode. The commercial Ag/AgCl pastes are composed of polymer resins such as polyvinyl butyral (PVB), which facilitates maintaining a stable potential over a long period of time in samples with different ionic strengths [17]. A photograph of the fabricated sensors on two glass substrates are shown in Figure 1. The pH, free Cl and BPA sensors are fabricated on one glass microscope slide, whereas the temperature sensor is fabricated on another glass slide. The two glass slides are then placed slide-by-side and connected to the readout printed circuit boards (PCB). The sensors are cleaned with tap water and stored in a dry place when not in use.

### 2.2. Readout System and Smartphone Application

The MWQMS readout circuit board was developed on top of an Arduino Uno R3 (ATmega328P) 8-bit microcontroller. Additionally, a Water Quality Monitor (WQM) board and a potentiostat board was vertically staked on top of the Arduino board, as shown in Figure 1. The WQM and potentiostat boards contain the circuits associated with analog and mixed signal sensors. The WQM board was created and programmed to read pH and temperature data in real time (e.g., one second interval), and free chlorine every 50 s. The potentiostat board was intended to operate independent voltametric sensing using the smartphone application. A Bluetooth transceiver was also connected to the microcontroller unit for data transmission wirelessly to our custom app on an Android smartphone. The Android app contains two main units that communicate with the WQM and potentiostat circuit boards.

The WQM module can display real-time pH, free Cl and temperature data, and it also has settings options to input calibration information. The potentiostat module can run, save or create different types of voltametric sensing experiments. The total amount of time needed to run a single voltametric sensing experiment (i.e., BPA sensing) is the sum of pre-treatment time and scanning time (i.e., scan-rate × potential-range). The WQM and the potentiostat PCB boards are designed separately because of the fundamental difference of the sensor’s operation. In the WQM board, the signal from the sensor is transmitted only in one direction, which is from the sensors towards the PCB. On the other hand, the potentiostat board has two-way communication, in which a sweeping potential is being applied towards the electrochemical sensor, and the resulting voltametric current signal is being read almost at the same time, while it is being transmitted towards the potentiostat PCB. Because of these differences in sensor operation, the WQM and potentiostat cannot run at the same time.

## 3. Results

### 3.1. Design of the MWQMS System

The MWQMS system is designed for simultaneous in situ pH, free Chlorine (Cl) and temperature measurement, as well as click-on-demand BPA measurement in water. The fabrication of the sensors was based on two microscope glass slides, which offered an inexpensive sensors integration, as shown in Figure 1. The printed circuit boards (PCBs) were stacked together to integrate signal conditioning, processing and wireless data transmission on the same platform. This was possible with the ubiquitous and affordable integrated-circuits (ICs) and optimization of software and hardware designs [28]. The water quality parameters (i.e., pH and free Cl, temperature and BPA) were proposed to estimate the overall condition and drinkability of the water being tested. For instance, accurate monitoring of free Cl concentration is critical for the safety of public health, which is also advised by the World Health Organization (WHO). In contrast, free Cl concentration depends on the pH of water. Also, free Cl, and pH of water both depend on temperature. Therefore, temperature measurement is also important to compensate for the pH and free Cl sensor measurements. The selection of BPA was due to the increased presence of emerging organic micropollutants in water cycles, which may become a huge water problem worldwide, therefore requiring a user-friendly and inexpensive detection technique. Thus, the electrochemical sensing of BPA was chosen as an example of the versatile nature of our proposed MWQMS system.

### 3.2. Signal Flow and PCB Design

**Figure 2:**
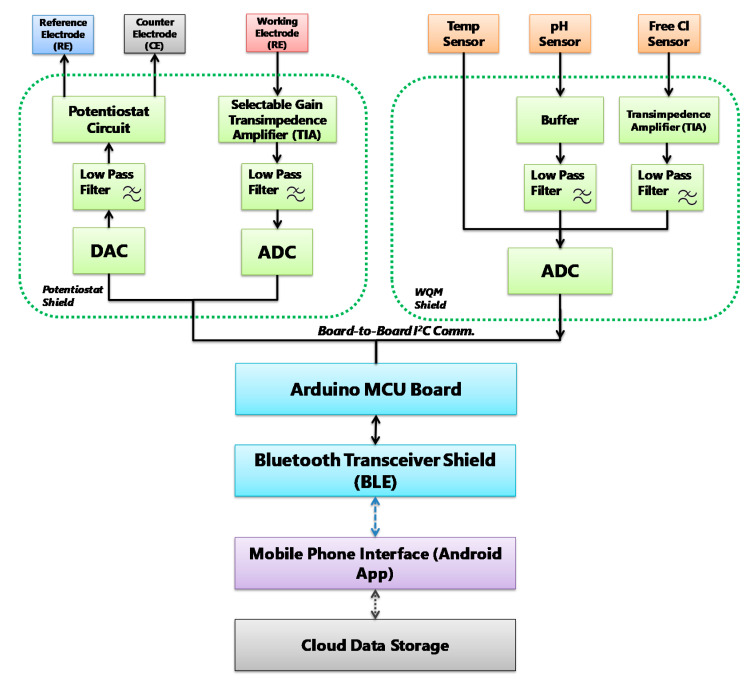
MWQMS system block diagram of the signal flow path. Each unit is color coded. For example, signal transduction unit: orange, signal conditioning unit: green, signal processing unit: blue, and wireless transmission unit: blue paths and Android application: purple.

The design of the signal flow of sensors is an important step towards designing suitable printed circuit boards (PCBs) for sensor data acquisition and interfacing. Figure 2 illustrates the block diagram of the signal flow paths that consist of signal transduction, conditioning, processing and wireless transmission routes. The signal flow is designed to facilitate simultaneous (pH, free Cl and temperature) and click-on-demand (BPA) monitoring of these water quality parameters. In summary, this diagram shows the signal-conditioning route for every single sensor, which are employed with analogue circuit components such as low-pass filter, buffer, transimpedance amplifier, and potentiostat corresponding to the transduced sensor signals.

**Figure 3:**
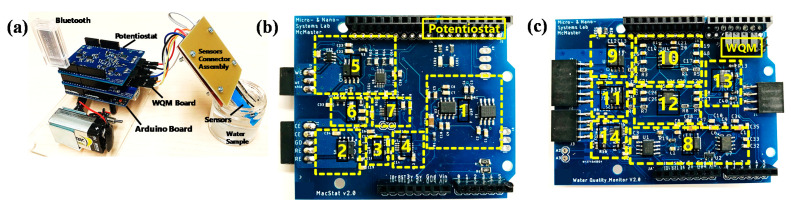
(**a**) Photograph of the MWQMS system with the sensors. Top view of (**b**) the potentiostat shield, and (**c**) the WQM shield. The numbered yellow boxes refer to the different integrated circuit components. (1 & 8) Power Supply, (2) potentiostat Circuit, (3) LPF (DAC output filter), (4) DAC, (5 & 11) TIA, (6) LPF (ADC input filter), (7) ADC, (9) Buffer, (10) LPF (pH), (12) LPF (Cl), (13) ADC (Cl + pH), (14) ADC (Temperature).

The circuits are designed to ensure fine resolution of the sensor signal while keeping the signal amplitude in the input range of the analogue-to-digital converter (ADC). After that, the conditioned signals are compensated, and relayed to the Bluetooth wireless transceiver by the serial communication protocols of the Arduino microcontroller. The Bluetooth transceiver facilitates wireless data transmission to a Bluetooth-supported smartphone and a custom-designed app. The smartphone application consists of an interface to upload sensor measurement data to online storage. The signal flow diagram was used to design the PCBs of the MWQMS system. For example, the upper-left, upper-right and lower blocks of the signal flow (Figure 2) diagram represent the potentiostat, the WQM and the Arduino Uno microcontroller PCBs, respectively. A photograph of the MWQMS system is shown in Figure 3a, depicting the PCBs that are connected to the sensors through a sensor connection assembly.

The potentiostat and WQM PCBs are shown in detail in the photographs of Figure 3b,c, respectively. The size of the PCBs is designed with respect to the size of an Arduino Uno microcontroller, so that they can be vertically stacked easily. This approach significantly reduced the overall size of the system. The major functional circuit components are shown with yellow dashed rectangles. Each PCB consisted of their own power supply unit (1 and 8). The potentiostat PCB consisted of a potentiostat Circuit IC (2), a low-pass filter (LPF) (3) connected to digital-to-analog converter (DAC) (4) output filter, a transimpedance amplifier (TIA) (5), and another LPF (5) connected to an ADC input filter (7). The WQM PCB consisted of buffers (9), an LPF for the pH sensor (10), a TIA (11) and LPF (12) for the free Cl sensor, an ADC IC for both the pH and free Cl sensors (13), and an ADC for the temperature sensor (14).

**Figure 4:**
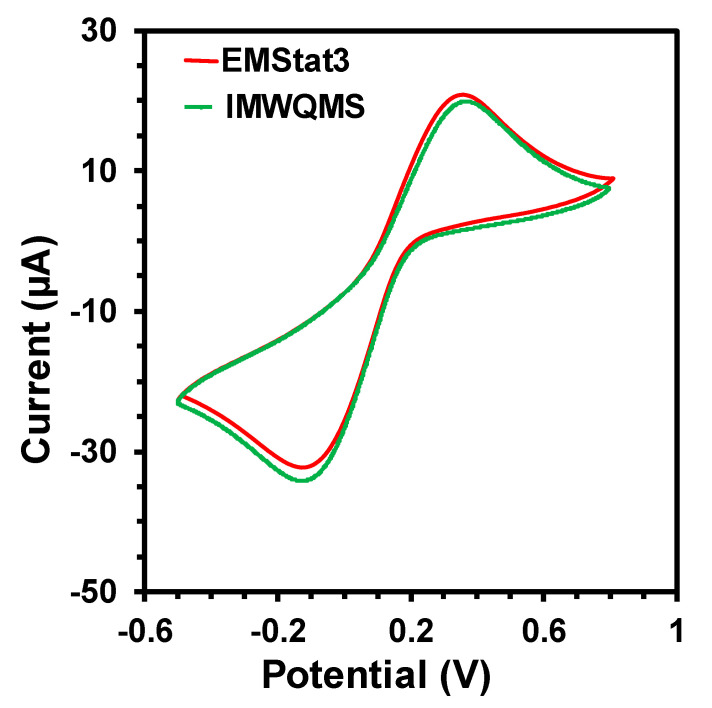
Cyclic Voltammetry curves of an SPE electrode with 5 mM K_3_[Fe(CN)_6_] using commercial EMStat3 potentiostat and our developed MWQMS system.

The signal quality of the potentiostat was verified by comparing the cyclic voltammetry (CV) measurement of a standard redox probe (5 mM concentration of K_3_[Fe(CN)_6_]) with a screen printed carbon electrode (SPE) obtained from CH Instruments Inc. with EMStat 3 potentiostat from PalmSens.com. The comparative CV curves are shown in Figure 4. The curves show that the CV obtained from the MWQMS system is very similar to the one obtained from EMStat 3, as the redox peak positions and peak intensities are almost the same. There is a slight vertical shift of the curves, which corresponds to a shift in the baseline. However, the baseline shift did not change the peak positions and their absolute intensities. Such variations are very common in electrochemical measurements, as the two curves are obtained from two different measurement with two devices and one sample. The signal-to-noise ratio of the CV curves obtained from the MWQMS system is also very high (>100).

### 3.3. Sensor Performance and Calibration

#### 3.3.1. pH Sensor

**Figure 5:**
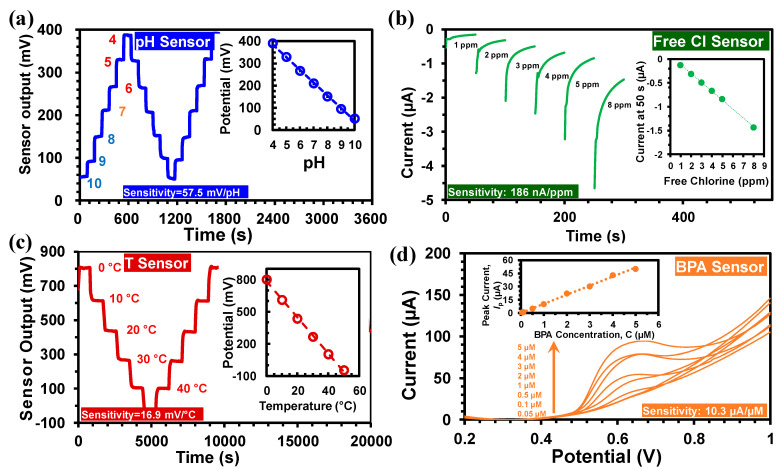
(**a**) The output of the pH sensor between pH 4 and 10, (inset) calibration curves. (**b**) Free chlorine sensor output with concentrations between 1 and 8 ppm, (inset) calibration curves. (**c**) The temperature sensor output between 0 °C and 50 °C, (inset) calibration curve. (**d**) Linear Sweep Voltammetry (LSV) of GO-MWCNT-βCD(SE)/SPE in 0.01 M PBS (pH 7.4) with 0.05 μM–10 μM BPA, (inset) calibration curve for BPA.

The Pd/PdO based potentiometric pH sensor had high sensitivity (57.5 mV/pH) and stability, as shown in Figure 5a [26]. The pH measurement range for calibration was from pH 4 to 10; however, the sensor is capable of measuring pH from 2 to 12. Also, the spin-coating fabrication required a very small amount (<10 µL) of the Pd ink precursor solution resulting in a lower cost (<10 cent). The pH sensing was based on the following redox reaction [4]:

(1) $$\mathrm{PdO}+2H_{3}O^{+}+2e^{-}↔\mathrm{Pd}+3H_{2}O,$$

The redox potential is determined using the Nernst equation:

(2) $$E=E_{0}-\frac{2.303RT}{F}\mathrm{pH},$$

where *E*_0_ is the standard electrode potential, *R* is the gas constant (8.31 J/mol/K), *T* is the absolute temperature and *F* is Faraday’s constant (96,485.33 C/mol). The sensor’s output voltage was between 0–400 mV, stable and with low-noise, allowing direct interfacing with the input of the ADC. The fast response time (~20 s) of the sensor enabled real-time pH monitoring. The MWQMS system can perform single-point (pH 7) and three-point calibration (at pH 4, 7, and 10) to accommodate sensors with different sensitivity and linear range. The temperature dependence of the pH sensor was compensated using [26,29]:

(3) $$\mathrm{pH}=7+\frac{E_{cal}-E_{meas}}{57.5+\left(T_{meas}-27\right)\times 0.22},$$

Finally, Equation (3) was programmed into the MWQMS system, where *E_cal_* is the recorded voltage (in mV) while the sensor uses calibration solution of pH = 7, *E_meas_* is the measured voltage (in mV) throughout pH sensing, and *T_meas_* (in °C) is the temperature of the water. The pH sensor resolution was 0.17 pH which was calculated from its hysteresis value of 9.8 mV [26]. The pH sensor showed negligible interference with common ions as discussed in our previous study [26].

#### 3.3.2. Free Cl Sensor

The free chlorine sensor was fabricated by amine-modification of carbon electrode [30]. The amperometric free Cl sensing involves electrochemical reduction of HOCl (free Cl) corresponding to the following chemical reaction:

(4) $$\mathrm{HOCl}+2e^{-}\to {\mathrm{Cl}}^{-}+{\mathrm{OH}}^{-},$$

The resultant current value (at 50 s in each measurement) is related to the HOCl concentration [26]. The HOCl concentration is subsequently employed to measure the free chlorine concentration (both HOCl and OCl^−^) that corresponds to the Cl_2_ concentration [30]. As shown in Figure 5b, the sensor output current range was between 0 and −5 µA, with free chlorine concentrations of 1 to 8 ppm. The sensor output was transformed into a voltage signal that ranged between 0 to 1 V utilizing a transimpedance amplifier. A low-pass filter was used to suppress the low-frequency noise in the transimpedance amplifier. The sensitivity of the free Cl sensor was 186 nA/ppm (Figure 5b). The temperature dependence of the free chlorine sensor was determined to be 6.2 nA/ppm/°C, which was used in the calibration equation of the free chlorine sensor [26]:

(5) $$I_{out}=\left[186+\left(T_{meas}-27\right)\times 6.2\right]C_{NaOCl}+64.7,$$

where *I_out_* (in nA) represent the output current, *T_meas_* (in °C) represent the temperature of water, and *C_NaOCl_* (in ppm) represent NaOCl concentration. The transfer function was calculated based on the transimpedance amplifier gains and low-pass filter as follows:

(6) $$V=0.196I_{out}+35.4,$$

where *V* (in mV) represent the output voltage of the signal conditioning circuit. Lastly, the free chlorine concentration equation with pH and temperature corrected calibration equation was programmed into MWQMS system [26]:

(7) $$\begin{array}{l} C_{free\;chlorine}=0.57\times \frac{\frac{V-35.4}{0.196}-64.7}{186+\left(T_{meas}-27\right)\times 6.2}\times \\ \left\{1+10^{pH-\left[\frac{3000}{T_{meas}+273}-10.0686+0.0253\left(T_{meas}+273\right)\right]}\right\}. \end{array}$$

The free chlorine sensor resolution was calculated by changing the free chlorine concentration between 1 and 8 ppm and measuring the hysteresis. The hysteresis value was estimated to be 11 nA equivalent to a resolution of 0.06 ppm [26].

#### 3.3.3. Temperature Sensor

The temperature sensor was developed based on a Wheatstone bridge configuration. Among the four arms of the bridge, two were made of p-type silicon wafer (1 cm × 10 cm) with a positive TCR of 1%/°C and two were fabricated by drop-casted PEDOT:PSS films with a negative TCR of −0.32%/°C [26,31,32]. The high TCR values of the silicon and PEDOT:PSS film provided high temperature sensitivity with negligible drift. The resistances of the four resistors were selected in such a way that the output voltage remains within the ADC input range of −1 to +1 V, towards measuring 0 °C–50 °C. The resistances were also optimized for highest sensitivity, reduced self-heating-induced drift, and the overall reduction of the area of the sensor. The measured sensitivity of the temperature sensor was 16.95 mV/°C as shown in Figure 5c. A calibration equation was used in the MWQMS system for the determination of temperature as follows [26]:

(8) $$T_{meas}=\frac{786.38-V_{out}}{16.95},$$

where *T_meas_* (in °C) represent the temperature of water and *V_out_* (in mV) represents the temperature sensor output voltage.

#### 3.3.4. BPA Sensor

The fabricated BPA sensor was based on a screen-printed electrode (SPE), which was integrated on the same glass substrate of the pH and free Cl sensor, followed by drop-cast modification with GO-MWCNT-βCD. The GO-MWCNT-βCD solution was prepared based on our previous study [27]. Briefly, MWCNTs were covalently modified with βCD through a one-step Steglich esterification method. Then, a 2 mg/mL MWCNT-βCD suspension was combined with 1 mg/mL GO suspension with a 1:1 volume ratio. After that, the solution was ultrasonicated for 15 min. Due to contamination of the electrode surface with residues of oxidized BPA, a freshly prepared GO-MWCNT-βCD/SPE electrode was used only once for BPA sensing. However, the SPE electrode was reused by cleaning the GO-MWCNT-βCD(SE) with cleaning solvents and re-drop-casting GO-MWCNT-βCD solution on the SPE working electrode. Therefore, the SPE electrode is reusable for multiple BPA sensing experiments, which can significantly decrease the cost of the sensor. A typical BPA sensing experiment in this study requires ~30 min, in which ~30 min is required for electrode pre-treatment (magnetic stirring of samples) and ~30 s is required for the actual voltametric potential sweeping [33].

Figure 5d shows the BPA sensing characteristics using the GO-MWCNT-βCD(SE)/SPE electrode with the MWQMS system. The BPA sensing was performed by Linear Sweep Voltammetry (LSV) from 50 nM to 5 µM BPA. The inset of Figure 5d illustrates the linear calibration curve for BPA with a slope of 10.3 µA/µM. The limit of detection (LoD = 3 *s*/*m*), was calculated to be 6 nM based on our previous study [27]. In this LoD calculation, *s* represents the standard deviation of the blank solution (30 nA) and *m* represents the slope of the calibration curve.

It is worth mentioning that a significant novelty of the MWQMS system stems from the integration of GO-MWCNT-βCD(SE)/SPE electrode with a custom-designed potentiostat board, that resulted in a very inexpensive and easy-to-use system for water monitoring. In addition to BPA sensing, the same electrochemical sensor can also be used for detecting pharmaceutical contaminants such as acetaminophen and estrogen, and heavy metal such as lead and arsenic, which can be accomplished by simply using different voltametric parameters in the potentiostat. This allows the MWQMS system to operate in a multi-modal sensing capability that is very cost-effective.

### 3.4. Smartphone Application

**Figure 6:**
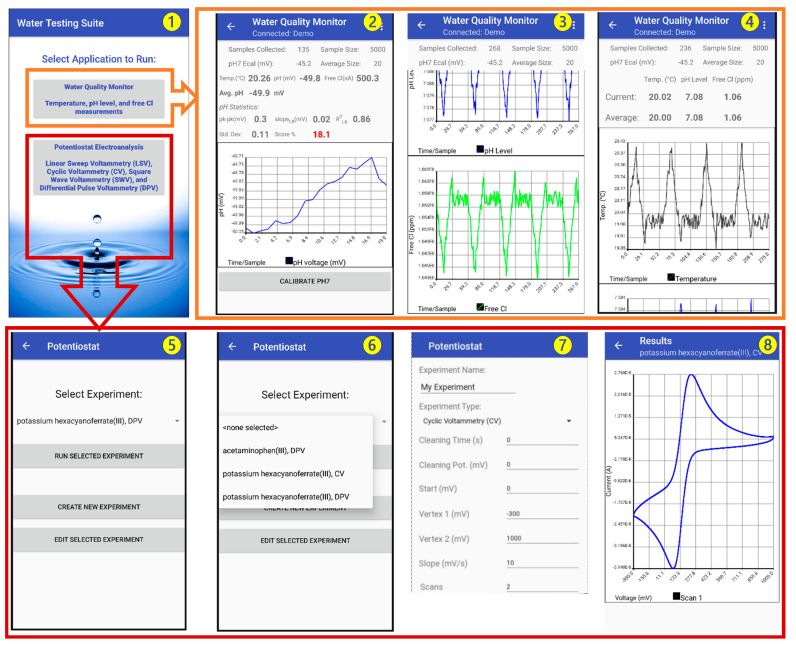
Screenshots of the MWQMS Android app on a Smartphone. Screenshot ❶: opening interface of the application. Screenshot ❷: temporal pH sensor data. Screenshot ❸: temporal free chlorine sensor data. Screenshot ❹: temporal temperature sensor. Screenshot ❺: potentiostat experiment option. Screenshot ❻: potentiostat experiment dropdown menu. Screenshot ❼: example of creating a CV experiment. Screenshot ❽: an example of CV curve.

An Android-based smartphone application called “Water Testing Suite” was designed for the MQWMS system. The application was first developed in Java and then compiled into an Android application. The application is designed to acquire the sensor data, perform calibration, and display and save the data in a cloud-based webserver. Figure 6 shows screenshots of different measurement units of the application. The opening screen of the application (screenshot 1) prompts the user to select the type of measurement to Run, such as Water Quality Monitor (for pH, free Cl and Temperature sensing), and potentiostat electroanalysis (for BPA sensing).

The selection of the Water Quality Monitor option in the opening screen leads to a screen containing a real-time display of measurement parameters for pH, free Cl and temperature of the water sample (screenshot 1). This screen also contains three graphs (to be scrolled down) for the corresponding sensor outputs. Therefore, this screen provides real-time update of the temporal measurement data. The sensor output is updated every second and the historical data is accumulated in the temporal graphs.

The pH sensor output graph (screenshot 2) can be calibrated using an on-screen one-point calibration button at a pH 7. This allows faster calibration of the pH sensor when the sensitivity of the sensor is very stable. However, direct calibration can also be done by going to the “Settings” option, where the user can put calibration information in terms of the sensor sensitivity and *y*-axis intercept potential values. This enables the ubiquitous nature of the MWQMS system to be used with other types of potentiometric pH sensors. The free Cl and temperature sensor output graphs (screenshots 3 and 4) are also updated at the same time with the pH sensor every second. The pH and temperature sensor data are used to calibrate the free Cl concentration measurement.

The selection of the Potentiostat Electroanalysis option in the opening screen leads to a screen containing options (screenshot 5) to Run, Edit or Create a new voltametric measurement, such as Linear Sweep Voltammetry (LSV), Cyclic Voltammetry (CV), Differential Pulse Voltammetry (DPV) and Square Wave Voltammetry (SWV) (screenshot 6). A selection of previously saved experimental parameters can be accessed from a drop-down menu, as shown in screenshot 6. A new experiment with custom parameters can also be created, as shown in screenshot 7. A representative CV curve is shown in screenshot 8.

### 3.5. Drift and Interference

**Figure 7:**
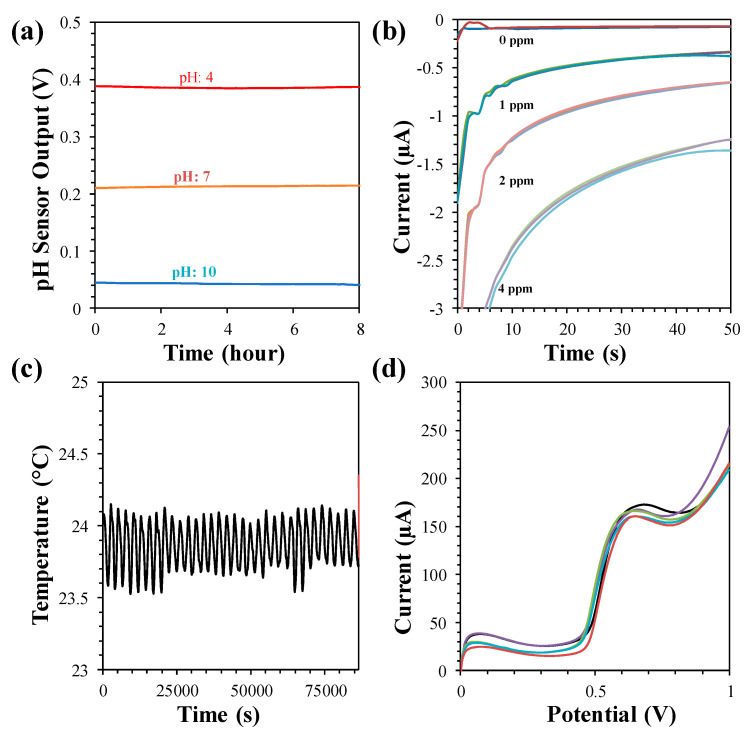
(**a**) Drift of pH sensor at pH 4, 7 and 10 measured in 8 h. (**b**) Drift of free chlorine sensor at 0, 1, 2 and 4 ppm measured once in every hour for three times in each concentration. (**c**) Drift of temperature sensor measured at 23.8 °C for 24 h. (**d**) Interference behavior of BPA sensor with 5 µM BPA.

The pH sensor does not need calibration for a continuous measurement of one hour. The drift of the pH sensor was investigated in our previous study, which showed a drift of 4 mV in pH 4 in an 8 h period that corresponded to a drift of 0.009 pH/h, as displayed in Figure 7a. The interference of the pH sensor was determined in the presence of interfering ions (CaCl_2_, KNO_3_, Na_2_(SO_4_), (NH_4_)_2_SO_4_, NaCl, and KCl). A maximum of a 0.24 pH change was observed with 0.1 M CaCl_2_ that was, in fact, due to transformation of the sample pH with the high concentrations of CaCl_2_.

The temporal response of the free chlorine sensor also showed negligible drift (<0.1 ppm), as shown in Figure 7b. The selectivity of the free chlorine sensor was also investigated for 2 ppm free chlorine solutions with ~400 ppm of interfering ions. A negligible change was observed with the interfering solution of KNO_3_, Na_2_(SO_4_), NaCl, and KCl. However, the free chlorine response became 0 ppm when CaCl_2_ and (NH_4_)_2_SO_4_ were added, as they chemically reacted with the free chlorine. The time-dependent drift performance of the temperature sensor was studied. The drift study showed negligible drift of ~0.6 mV over a 24 h period, as indicated in Figure 7c. The high selectivity of the BPA sensor was also demonstrated with other interfering species such as Na^2+^, K^+^, ascorbic acid, dopamine, and acetaminophen, as shown in Figure 7d.

The lifetime of the sensors was studied by performing long-term analysis with at least 6-month old sensors. A 7 day long measurement of pH, free chlorine, and temperature of real water samples taken from a lake, tap and pool was done by the MWQMS system [26]. The pH sensor showed a highest drift of 0.12 pH for tap water. The temperature sensor also showed a drift of ±0.25 °C. The free chlorine sensor showed gradual decay of free chlorine due to interaction with the sensor as well as exposure to the external environment.

### 3.6. Real Sample Analysis

The MWQMS was used to measure real samples from tap water, lake water, and swimming pool water. A comparison of the pH, free chlorine, and temperature measured by the MWQMS system and using reference methods are shown in Table 1. A commercial glass electrode-based pH meter (HANNA HI98128) was utilized as the reference for pH and temperature sensing. A commercial DPD-based kit (LaMotte 2056 ColorQ PRO 7) was used as a reference for free chlorine sensing. Table 2 shows the difference of the BPA measurements done by the MWQMS system after spiking tap water with BPA. The measurement results of the MWQMS system were closely comparable to those of the commercial methods. Differences below 5% were observed for pH, free chlorine, and temperature sensors, while the BPA measurements showed recovery between 96% and 106%. The differences in our measurement and reference values are, therefore, reasonable in practical application scenarios. These differences may be attributed to errors/deviations in the commercial pH, free chlorine and temperature sensors themselves, or human errors.

### 3.7. Limitations and Future Improvements

The MWQMS system can be further improved by tackling some of its limitations that are related to both the sensors and readout systems. For example, the sensors were mostly hand-made by lab-oriented fabrication methods. However, these methods can be tailored for mass production such as screen-printing and solution processing, for scaling up the fabrication and reducing overall costs of the sensor. Such processes will improve sensor reproducibility and thus sensing performance. Also, reusability of the sensors can be improved by careful choice of sensing materials and sensing methods. Further, the readout system used the Arduino Uno based microcontroller, which can be re-designed with smaller microcontroller unit to reduce the footprint of the system towards a hand-held device configuration.

## 4. Conclusions

We developed an integrated multi-parameter water quality monitoring system (MWQMS) that can concurrently determine water parameters such as pH, free chlorine concentration and temperature, as well as determine bisphenol A on-demand. The MWQMS system was comprised of a Pd/PdO-based pH sensor, a carbon-based free chlorine sensor, a hand-drawn temperature sensor, and a graphene oxide, carbon nanotubes and β-cyclodextrin bisphenol A sensor, all of these fabricated on two glass slides. The sensors can measure water quality parameters such as pH and temperature in real time, free chlorine in every 50 s, and BPA in half an hour, with a high sensitivity of 57.5 mV/pH (pH), 186 nA/ppm (free chlorine), 16.95 mV/°C (temperature) and 10.3 µA/ppm (bisphenol A), in a user-friendly manner. The MWQMS system is small and a simple-to-use measurement unit with a smartphone application. It is a promising step towards practical applications such as on-site water quality monitoring. The system also has the flexibility to be modified to accommodate additional water quality monitoring sensors including conductivity, dissolved oxygen and different types of ions. Finally, improvements are already underway in our ongoing research efforts towards commercialization of the water quality monitoring system and more extensive testing in application environments.

## Figures and Tables

**Table 1:** Real water sample measurement for pH, free chlorine concentration and temperature (average determined from 5 measurements).

	Tap Water (Hamilton)		Lake Water (Lake Ontario)		Pool Water (Hamilton)	
	MWQMS	Ref.	MWQMS	Ref.	MWQMS	Ref.
**pH**	8.0 ± 0.1	8.2 ± 0.1	7.4 ± 0.1	7.5 ± 0.1	7.1 ± 0.2	7.2 ± 0.1
**Free Cl, ppm**	1.1 ± 0.1	0.95 ± 0.05	0.00	0.00	2.6 ± 0.1	2.4 ± 0.1
**Temp., °C**	15.6 ± 0.2	15.3 ± 0.1	14.5 ± 0.2	14.8 ± 0.1	24.6 ± 0.2	24.8 ± 0.1

**Table 2:** Measurement results of BPA in spiked tap water using GO-MWCNT-βCD(SE)/SPE (Average determined from 5 measurements).

	Prepared Concentration (µM)	Found (µM)	Recovery (%)
**BPA**	0.50	0.48 ± 0.02	96.0 ± 4.2
	1.00	1.06 ± 0.03	106.0 ± 2.8
	5.00	4.97 ± 0.10	99.4 ± 2.0

## Data Availability

Not applicable.

## References

[1] World Health Organization (2011). Guidelines for Drinking-Water Quality.

[2] Chapman D. (1992). Water Quality Assessments.

[3] Storey M.V., van der Gaag B., Burns B.P. (2011). Advances in on-line drinking water quality monitoring and early warning systems. Water Res..

[4] Qin Y., Alam A.U., Pan S., Howlader M.M.R., Ghosh R., Selvaganapathy P.R., Wu Y., Deen M.J. (2016). Low-temperature solution processing of palladium/palladium oxide films and their pH sensing performance. Talanta.

[5] Alam A.U., Qin Y., Nambiar S., Yeow J.T.W., Howlader M.M.R., Hu N.-X., Deen M.J. (2018). Polymers and organic materials-based pH sensors for healthcare applications. Prog. Mater. Sci..

[6] USEPA (2005). Water Sentinel Online Water Quality Monitoring as an Indicator of Drinking Water Contamination.

[7] Piranti A., Waluyo G., Rahayu D.R.U.S. (2019). The possibility of using Lake Rawa Pening as a source of drinking water. J. Water Land Dev..

[8] Qin Y., Kwon H.-J., Howlader M.M.R., Deen M.J. (2015). Microfabricated electrochemical pH and free chlorine sensors for water quality monitoring: Recent advances and research challenges. RSC Adv..

[9] First Nations Food, Nutrition & Environment Study, Results from Ontario. http://www.fnfnes.ca/docs/FNFNES_Ontario_Regional_Report_2014_final.pdf.

[10] Liao C., Kannan K. (2011). Widespread Occurrence of Bisphenol A in Paper and Paper Products: Implications for Human Exposure. Environ. Sci. Technol..

[11] Banna M.H., Imran S., Francisque A., Najjaran H., Sadiq R., Rodriguez M., Hoorfar M. (2014). Online drinking water quality monitoring: Review on available and emerging technologies. Crit. Rev. Environ. Sci. Technol..

[12] Joo S., Brown R.B. (2008). Chemical sensors with integrated electronics. Chem. Rev..

[13] Feng Y., Smith D.W., Bolton J.R. (2007). Photolysis of aqueous free chlorine species (HOCl and OCl^−^) with 254 nm ultraviolet light. J. Environ. Eng. Sci..

[14] Badihi-Mossberg M., Buchner V., Rishpon J. (2007). Electrochemical Biosensors for Pollutants in the Environment. Electroanalysis.

[15] Lee H., Choi T.K., Lee Y.B., Cho H.R., Ghaffari R., Wang L., Choi H.J., Chung T.D., Lu N., Hyeon T. (2016). A graphene-based electrochemical device with thermoresponsive microneedles for diabetes monitoring and therapy. Nat. Nanotechnol..

[16] Nyein H.Y.Y., Gao W., Shahpar Z., Emaminejad S., Challa S., Chen K., Fahad H.M., Tai L.-C., Ota H., Davis R.W. (2016). A wearable electrochemical platform for noninvasive simultaneous monitoring of Ca^2+^ and pH. ACS Nano.

[17] Gao W., Emaminejad S., Nyein H.Y.Y., Challa S., Chen K., Peck A., Fahad H.M., Ota H., Shiraki H., Kiriya D. (2016). Fully integrated wearable sensor arrays for multiplexed in situ perspiration analysis. Nature.

[18] Xu Z., Dong Q., Otieno B., Liu Y., Williams I., Cai D., Li Y., Lei Y., Li B. (2016). Real-time in situ sensing of multiple water quality related parameters using micro-electrode array (MEA) fabricated by inkjet-printing technology (IPT). Sens. Actuators B Chem..

[19] Mross S., Zimmermann T., Winkin N., Kraft M., Vogt H. (2016). Integrated multi-sensor system for parallel in-situ monitoring of cell nutrients, metabolites, cell density and pH in biotechnological processes. Sens. Actuators B Chem..

[20] Kirsanov D., Korepanov A., Dorovenko D., Legin E., Legin A. (2017). Indirect monitoring of protein A biosynthesis in E.coli using potentiometric multisensor system. Sens. Actuators B Chem..

[21] Banna M.H., Najjaran H., Sadiq R., Imran S.A., Rodriguez M.J., Hoorfar M. (2014). Miniaturized water quality monitoring pH and conductivity sensors. Sens. Actuators B Chem..

[22] Zhou B., Bian C., Tong J., Xia S. (2017). Fabrication of a Miniature Multi-Parameter Sensor Chip for Water Quality Assessment. Sensors.

[23] Mamun K.A., Islam F.R., Haque R., Khan M.G.M., Prasad A.N., Haqva H., Mudliar R.R., Mani F.S. (2019). Smart Water Quality Monitoring System Design and KPIs Analysis: Case Sites of Fiji Surface Water. Sustainability.

[24] Park J., Kim K.T., Lee W.H. (2020). Recent Advances in Information and Communications Technology (ICT) and Sensor Technology for Monitoring Water Quality. Water.

[25] Pasika S., Gandla S.T. (2020). Smart water quality monitoring system with cost-effective using IoT. Heliyon.

[26] Alam A.U., Clyne D., Jin H., Hu N.-X., Deen M.J. (2020). Fully Integrated, Simple, and Low-Cost Electrochemical Sensor Array for in Situ Water Quality Monitoring. ACS Sens..

[27] Alam A.U., Qin Y., Catalano M., Wang L., Kim M.J., Howlader M.M.R., Hu N.-X., Deen M.J. (2018). Tailoring MWCNTs and β-Cyclodextrin for Sensitive Detection of Acetaminophen and Estrogen. ACS Appl. Mater. Interfaces.

[28] Jin H., Qin Y., Pan S., Alam A.U., Dong S., Ghosh R., Deen M.J. (2018). Open-Source Low-Cost Wireless Potentiometric Instrument for pH Determination Experiments. J. Chem. Educ..

[29] Qin Y., Alam A.U., Pan S., Howlader M.M.R., Ghosh R., Hu N.-X., Jin H., Dong S., Chen C.-H., Deen M.J. (2018). Integrated water quality monitoring system with pH, free chlorine, and temperature sensors. Sens. Actuators B Chem..

[30] Pan S., Deen M.J., Ghosh R. (2015). Low-Cost Graphite-Based Free Chlorine Sensor. Anal. Chem..

[31] Li S.S., Thurber W.R. (1977). The dopant density and temperature dependence of electron mobility and resistivity in n-type silicon. Solid State Electron..

[32] Kwon I.W., Son H.J., Kim W.Y., Lee Y.S., Lee H.C. (2009). Thermistor behavior of PEDOT:PSS thin film. Synth. Met..

[33] Alam A.U., Deen M.J. (2020). Bisphenol A Electrochemical Sensor Using Graphene Oxide and β-Cyclodextrin-Functionalized Multi-Walled Carbon Nanotubes. Anal. Chem..

